# RNase footprinting demonstrates antigenomic hepatitis delta virus ribozyme structural rearrangement as a result of self-cleavage reaction

**DOI:** 10.1186/1756-0500-1-15

**Published:** 2008-05-16

**Authors:** Larissa Savochkina, Victoria Alekseenkova, Tatyana Belyanko, Nadezhda Dobrynina, Robert Beabealashvilli

**Affiliations:** 1 Russian Cardiology Research and Production Center, 121552 Moscow, Russia

## Abstract

**Background:**

Hepatitis delta virus (HDV) is a satellite virus of hepatitis B. During viral replication the 1700-nucleotide-long genomic RNA and its complement, the antigenomic RNA, undergo self-cleavage catalyzed by internal ribozyme motifs that are essential for propagation of the virus *in vivo*. These self-cleavage activities are provided by 85-nucleotide-long sequence elements, the genomic and antigenomic forms of HDV ribozyme. Recently four permuted variants of the antigenomic HDV *cis*-ribozyme with a self-cleavage site located at the 5' proximity, in the middle, or nearby the 3' end of the molecule were constructed and synthesized. These constructs exhibit equal activity, a bi-phasic kinetics of self-cleavage reaction and reaction products with low and high stability. We have used ribonuclease probing to footprint the structures of uncleaved and post-cleaved forms of the antigenomic HDV ribozymes in solution. Uncleaved ribozymes, associated and individual products of the self-cleavage reaction were analyzed using ribonuclease and Fe(II)-EDTA protection assays to reveal the differences in the structure of pre- and post-cleaved antigenomic HDV ribozyme in solution.

**Findings:**

Our findings demonstrate that a significant conformational change accompanies catalysis in the antigenomic HDV ribozyme in solution, in contrast to minor conformational switch observed in crystals of the genomic form. This study indicates that changes in the structure of stem P1 and stem P4 are minor, those of the region ascribed to stem P2, stem P3 and loop l3 are dramatic, while stem P1.1 results from the self-cleavage reaction.

**Conclusion:**

Our data agree with the structure of post-cleaved and disagree with that of pre-cleaved forms of HDV ribozyme published elsewhere.

## Background

According to the double nested pseudoknot model, both genomic and antigenomic ribozymes fold into a similar secondary structure consisting of base-paired stems (P1 - P4), joining sequences (J1/2, J1/4, J4/2) and loops (l3, l4) [1]. Analysis the crystal structure of the 3' product of self-cleaved genomic HDV ribozyme showed, that the ribozyme adopts a tight tertiary structure by forming, in addition to the pseudoknot stems P1 - P4, the two-base-pair stem P1.1, thus creating a pair of nested pseudoknots, which buries the active site in a deep cleft [2,3].

However, when the crystal structure of the pre-cleaved form of *cis*-acting genomic HDV ribozyme was solved, it turned out that its structure differs from that of the product form, while the structure of stems P1–P4 and P1.1 is the same in both states [4].

**Figure 1:**
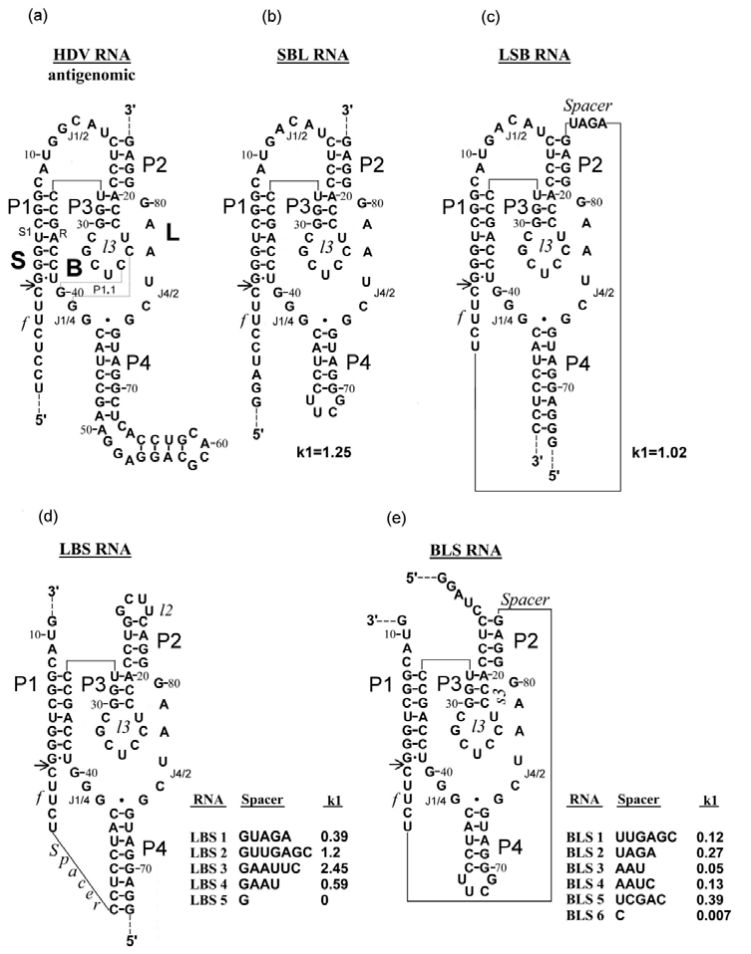
**Nucleotide sequence and proposed secondary structure of designed variants of HDV antigenome ribozyme**. (a) The wild type HDV antigenome ribozyme. (b)-(e) Variants of antigenome ribozyme: SBL (b), LSB (c), and the families LBS (d) and BLS (e). Inserts show the spacer sequence and activity (k1, min-1). The nucleotides are numbered according to Rosenstein and Been [7]. P2–P4, stems 2–4; P1.1, stem 1.1; s1, substrate containing part of stem P1; R, substrate recognition part of stem P1; J1/2, J1/4, J4/2, nucleotide sequences connecting stems P1 and P2, P1 and P4, P4 and P2; l2, l3 and l4, sequence connecting 5' and 3' sides of P2, P3 and P4; Sp (spacer), sequence connecting two ribozyme parts (L and S or B and S); f (forepart), an oligonucleotide which is always located at the 5'side of S chain. Only SBL topology corresponds to natural ribozyme.

We have prepared a set of permuted variants of HDV ribozyme with different locations of the cleavage site and different spacers connecting three major uninterruptible ribozyme parts (Figure 1). We analyzed the structures of all ribozyme constructs in pre- and post-cleaved states using RNAse and Fe(II)-EDTA footprinting.

Here we present the evidence that the conformations of pre- and post-cleaved antigenomic HDV ribozymes differ dramatically. From analysis of these differences it was concluded that stem P1.1 formed by nucleotides C25 and C24 with G40 and G41 does appear after the self-cleavage reaction.

## Structure design of permuted variants of antigenomic HDV ribozyme

We have prepared four out of six possible permuted variants of the HDV ribozyme. Spacers between L, B and S chains varied in length and nucleotide sequence, while the nucleotide sequences of the chains were invariable and corresponded to those of naturally occurring antigenomic HDV ribozyme. Figure 1 shows structure and rate constant of the first reaction phase (k1) for all constructs. Briefly, all ribozymes with optimal spacers are almost equally active under individual optimal conditions, while activation energy and optimal temperature vary from ribozyme to ribozyme with the sharpest temperature curve for LSB. In general, the activities of ribozymes belonging to the same family vary with the length and sequence of spacers. A very short spacer makes the ribozyme inactive.

For detailed analysis of the reaction kinetics of all topological variants described here see our previous paper [5] and our laboratory web site [6], where the reader can change the initial conditions and parameters of the reaction and observe resulting changes in the kinetic curves. For comparison with the model suggested earlier [5] we have inserted an additional stage that leads to irreversibility of the reaction.

The major difference between SBL (an isomer with "natural" chain connectivity) and the other constructs is the stability of the reaction product. Short and loosely bound 5'proximal fragment of SBL readily dissociates from the product complex while the products of the other constructs remain in the complex at temperatures up to 50°C–95°C [5]. This is also the major difference between the variants of HDV *cis*-ribozyme investigated here and the previously studied products of genomic and antigenomic ribozymes which lost the 5'proximal fragment after self-cleavage.

## Analysis of the secondary structure of pre-cleaved and post-cleaved antigenomic HDV ribozymes

The secondary structure of all constructs in pre- and post-cleaved state was probed with T1, U2, A and V1 ribonucleases. In addition, for all constructs electrophoretically purified long fragment of post-cleavage product was probed.

## Pre-cleaved ribozymes

**Figure 2:**
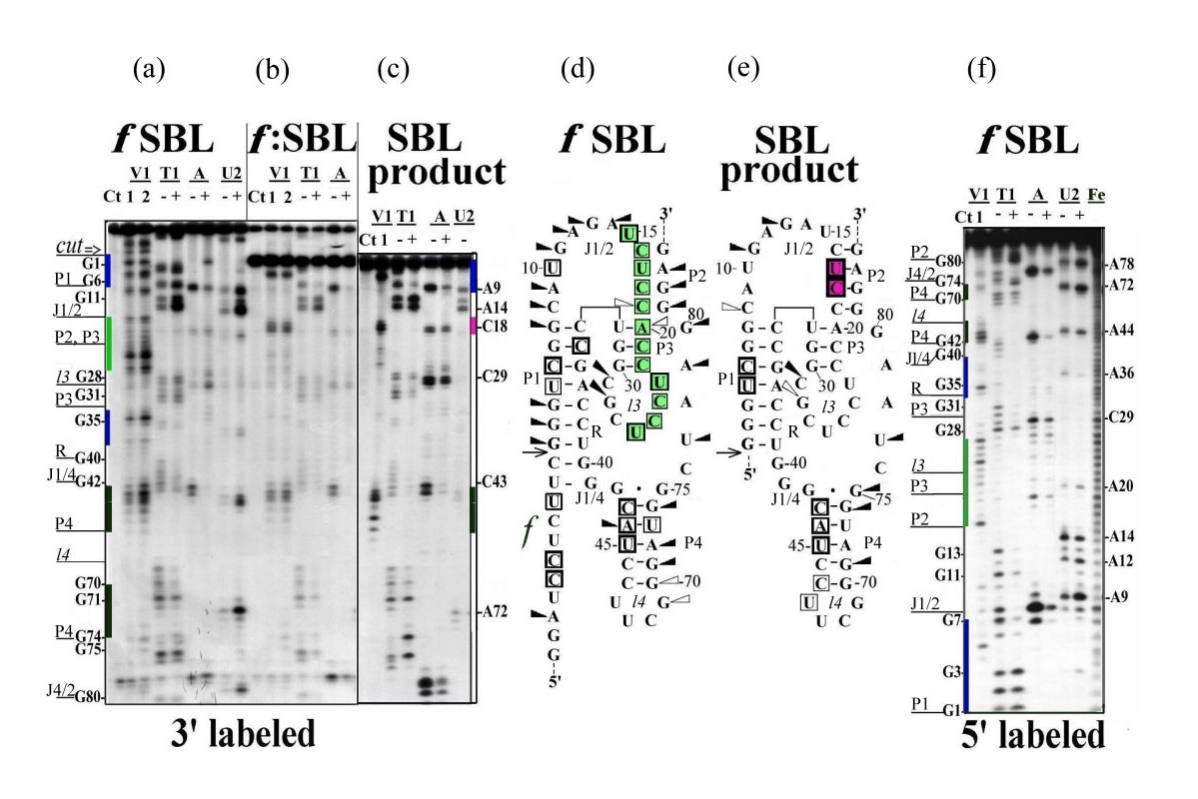
**RNase probing of SBL RNA**. (a)-(c) RNA labeled with ^32^P at the 3'-end or (f) at the 5'-end. (a) and (f) ^32^P labeled RNA was treated with nucleases under the conditions minimizing self-cleavage reaction (20°C, 5 min) or (b) self-cleaved at 50°C for 60 min and self-cleavage product was analyzed either directly in the mixture for self-cleaving or (c) after re-purifying by electrophoresis in denaturing polyacrylamide gel. The lanes marked with a plus sign are reactions carried out at 20°C, pH 7.5, 10 mM MgCl_2 _with either no added cleavage reagent (Ct) or with ribonuclease V1 (V1), ribonuclease T1 (T1), ribonuclease A (A) and ribonuclease U2 (U2). The minus sign lanes are reactions carried out at 50°C, pH 3.5 with 7 M urea (sequencing markers). Final concentration of RNases, reaction and analysis conditions were as described under Material and Methods (See Additional file 8). Two concentrations of RNase V1 were used: 1 × 10^-3 ^units/μl (1) or 5 × 10^-3 ^units/μl (2). V1 products contain a 3'-hydroxyl rather than phosphate, which results in decreased mobility relative to the sequencing markers in the case of 5'-labeled RNA and increased mobility in the case of 3'-labeled RNA. Fe(II)-EDTA cleavage (lane Fe) was done to produce RNA-ladder. Note that hydroxyl radical cleavage creates 5'-labelled fragments that are one nucleotide shorter than the sequencing markers. The results of experiments shown in Figures 2(a)-(c), (f) and of others not shown are summarized on the proposed secondary structures both for original (d) and self-cleaved (e) SBL RNAs, respectively. Cut sites of ribonucleases T1, A and U2 are designated as triangles, arrows indicate sites of self-cleavage, and squares indicate positions cleaved by V1 nuclease. Only the RNase-sensitive sites qualitatively judged to be strongly cut under the condition stated are shown. The relative intensity of the triangles and squares corresponds to the relative intensity of electrophoretic bands. Green color indicates stacking area before self-cleavage, raspberry color indicates stacking area which appears after self-cleavage. Blue and black colors indicate P1 and P4 stems, respectively.

**Figure 3:**
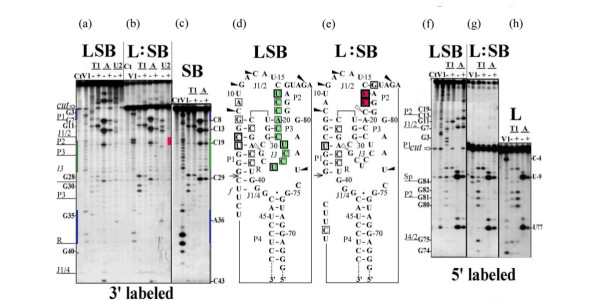
**RNase probing of LSB RNA**. (a)-(c) 3'-^32^P-labeled and (f)-(h) 5'-^32^P-labeled LSB RNA was treated with nucleases under different conditions: (a) and (f) 20°C, 5 min (minimizing self-cleavage); (b) and (g) 50°C, 60 min and self-cleavage product was analyzed either directly in the mixture or (c) and (h) after re-purifying by electrophoresis in denaturing polyacrylamide gel. Samples were analyzed as described for Figure 2 except that the concentration of V1 RNase was 1 × 10^-3 ^units/μl. Spacer nucleotides C-4 and U-9 were numbered in 3'-5'-direction from cut site and marked with "-" sign. The results of experiments shown in Figure 3(a)-(c) and 3(f)-(h) and of others not shown are summarized on the proposed secondary structures for pre-cleaved (d) and post-cleaved LBS RNA (e). Symbols used are as in Figure 2.

**Figure 4:**
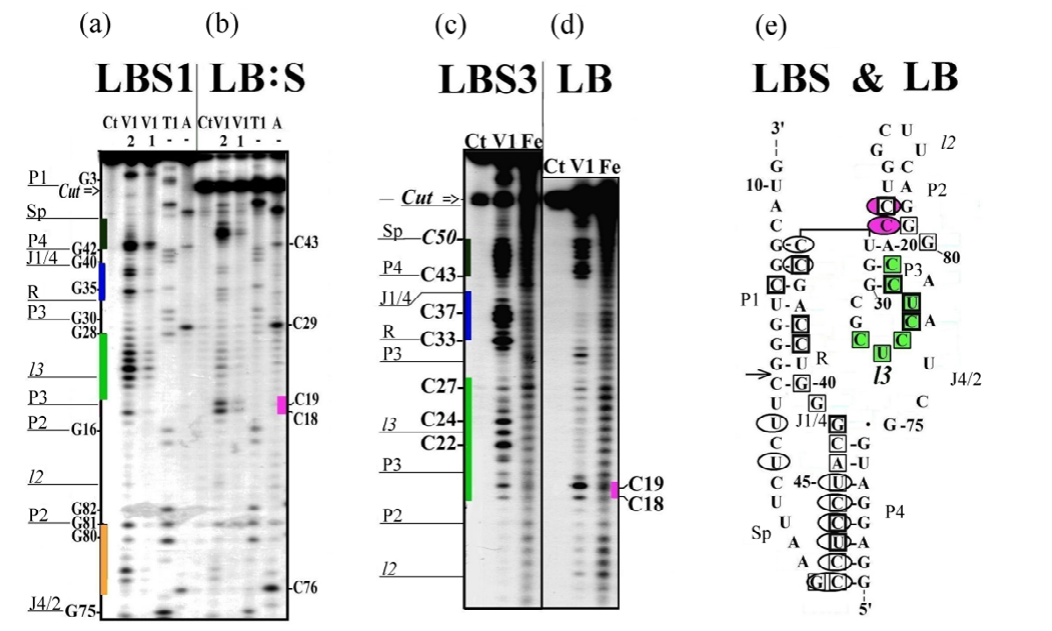
**Nuclease V1 and hydroxyl radical (Fe(II)-EDTA) cleavage of 5'-^32^P-labeled LBS1 and LBS3 RNAs**. (a) LBS1 was treated with nucleases under the conditions minimizing self-cleavage or (b) incubated at 50°C for 60 min and ^32^P-labeled product of self-cleavage was analyzed directly in the mixture. Samples (a) and (b) were analyzed as described in legend for Figure 2 except that for ribonucleases T1 and A only sequencing markers are shown. (c) LBS3 RNA and (d) its post-cleaved 5'-product re-purified by electrophoresis were subjected to ribonuclease V1 and Fe(II)-EDTA cleavage and analyzed as described under Material and Methods (See Additional file 8). (e) The results of experiments shown in Figures 4(a)-(d) and of others not shown are summarized on the proposed secondary structures for pre-cleaved and post-cleaved LBS RNA. Squares indicate the cut sites for V1 nuclease in pre-cleaved and ellipses in post-cleaved RNA. Only V1 nuclease-sensitive sites qualitatively judged to be strongly cut under the conditions stated are shown. The relative intensity of squares corresponds to the relative intensity of electrophoretic bands. Green color indicates stacking area before self-cleavage, raspberry color indicates stacking area which appears after self-cleavage. Blue and black colors indicate P1 and P4 stems, respectively.

All studied ribozymes which exhibit almost equal activity have similar nuclease digestion profiles for the original molecule (Figures 2a and 2f, 3a and 3f, 4a and 4c). The nuclease cleavage patterns are not the same, but the major features are similar. All joining sequences J1/2 in SBL and LSB, J4/1 in LBS and 3'-part of J2/1 in BLS (not shown) are susceptible to single-strand-specific nucleases but not to the double-strand-specific nuclease V1. Only individual weak V1 cuts are seen in joining sequences (at nucleotides (nt) U10 в SBL и A9 в LSB). The same is true for J4/2 for all pre-cleaved ribozymes of the LBS family (Figure 4a). The bottom of P4 and the ends of R are well accessible to double-strand-specific nuclease in all studied structures. In all constructs, the middle of R region is probably less accessible than the ends. In pre-cleaved ribozymes, the upstream regions of P2, P3 and l3 (between nt 15 and 26) are accessible to double-strand-specific nuclease. The relative intensities of different bands in this region are different for different ribozymes with slight preference for 15U, 23U and 26U in SBL (Figures 2a and 2f)) and 17U, 23U and 26U in LSB (Figure 3a). In LBS1, LBS3 (Figures 4a and 4c) and in LBS5, BLS1, BLS3 (not shown) the most accessible nucleotides are located between 22C and 25C. Digestion of P4 correlates with the activity of LBS. In the most active LBS3 (Figure 4c) the region is the most accessible to V1, in less active LBS1 it is only partially accessible (Figure 4a), while in inactive LBS5 the region is not accessible (not shown). This finding suggests that the shortening of joiner J4/1 to a single nucleotide prevents formation of P4 and makes the ribozyme inactive.

Note that pre-cleaved ribozyme exposed to nucleases at neutral pH in the presence of Mg2+ contains some post-cleavage products. The higher the activity of ribozyme, the greater the proportion of self-cleavage product in a pre-cleaved ribozyme preparation. This accounts for the presence of some minor bands typical of the product in the pattern of a pre-cleaved ribozyme. Vice versa, post-cleaved ribozymes are not fully cleaved and about 20% of uncleaved form is present in each preparation. Therefore, all bands belonging to uncleaved molecule are also present on autoradiograms with a five-fold lower intensity. Having this in mind, we considered only the relative intensities of the bands.

## Post-cleaved ribozymes

As already mentioned, V1 digestion pattern for pre-cleaved ribozymes shows well-defined bands with different but comparable intensities for each nucleotide between 15 and 26 nucleotides. This pattern is routine for each uncleaved construct. After cleavage, all these bands are replaced by a strong doublet at nt U17–C18 (SBL, LSB and BLS) or nt C18–C19 (LBS) (Figures 2b, 3b, 4b and 4d).

It is noteworthy that V1 digestion pattern of post-cleaved HDV ribozyme reported by Rosenstein & Been [7] also contains a strong doublet U17–C18 and is practically free from cut sites between nt 15 and 26. Interestingly, point mutations in the 3'product P2 region which disrupt P2 lead to a nuclease-digestion pattern typical of uncleaved unmodified ribozyme (cuts in the upstream strand of P2, P3 and l3 between position 16 and 26), while compensatory changes which restore stem P2 of 3'product provide a pattern typical of the 3'-product (cuts at positions 17 and 18) [8]. However, uncleaved LBS exhibits a pattern typical of the uncleaved ribozyme (cuts between position 18 and 26), irrespective of the presence of UUCG which closes nucleotide sequence and forces the formation of stem P2.

Purified large product of ribozyme self-cleavage also exhibits one of the two alternative states for the region between U15 and U26. Note that the product purification procedure includes unwinding the whole structure of RNA by a denaturant and subsequent annealing. After this procedure, the purified products of SBL (Figure 2c) and LBS (LB, Figure 4d) acquire a structure with a doublet at C18 region, while the product of LSB (SB, Figure 3c) regains the structure characteristic of the pre-cleaved form. It should be noted that the product of SBL self-cleavage possesses all elements included in the pseudoknot model. The product of LBS (LB) does not have the S region and therefore cannot form stem P1. The product of LSB (SB) cannot form stem P4 or stem P2 since it lacks L chain. Comparison of Figures 3a and 3c shows that the digestion patterns of the region between G1 and G40 for LSB and SB are almost identical except for very strong cleavages in R region of SB which are very weak in LSB. Thus, it can be suggested that in uncleaved ribozyme SB fragment is folded as a separate domain without direct interaction with P4 and L fragment. Previously we have shown that SB domain forms autonomously [9]. Obviously, stem P1 is present in SB and there is no candidate for forming a lengthy Watson-Creek double-stranded region with a polypyrimidine stretch from U17 to U26. However, the nucleotides of the region seem to be well stacked as they are well accessible to V1 nuclease and are not susceptible to single-strand-specific nucleases. In contrast to P2 and P3–l3, other parts of post-cleaved SBL and BLS exhibited no changes in nuclease digestion patterns.

It should be noted that the most unexpected changes occurred in the susceptibility of l2 to Fe(II)-EDTA cleavage (Figures 4c and 4d). According to any available 3D model of the ribozyme, this part of the molecule may become fully exposed to the solvate.

**Figure 5:**
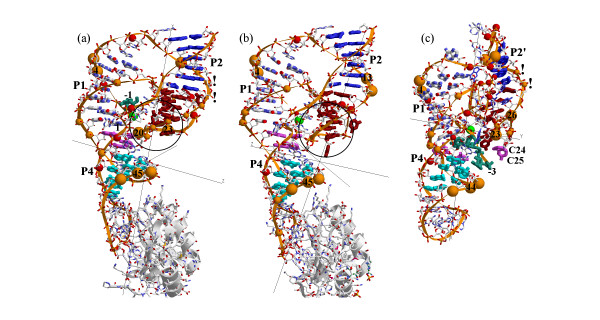
**RNase probing data for SBL ribozyme were projected on 3D model of HDV ribozyme**. (a) RNase probing data for pre-cleaved SBL ribozyme were projected on 3D model of pre-cleaved genomic HDV ribozyme; (b) RNase probing data for post-cleaved SBL ribozyme were projected on 3D model of the post-cleaved genomic HDV ribozyme; (c) RNase probing data for pre-cleaved SBL ribozyme projected on 3D model of the pre-cleaved SBL ribozyme. Red balls indicate cut sites for single-strand-specific nucleases, orange balls for double-strand-specific nuclease. Ball size is roughly proportional to the intensity of the corresponding lines on the electrophoregram. Green ball indicates the cut site for the self-cleavage reaction. Blue sticks denote nucleotide bases of stem P2 in (a) and (b) and stem P2' composed of nt 16–18 and nt 11–13 in (c). Cyan sticks nucleotide bases of stem P4, brown of stem and loop P3–l3, lilac of stem P1.1 and CPK of stem P1. Dark green sticks in (a) and (c) denote the f region of the SBL ribozyme. This region is absent from structure depicted in (b). Two exclamation marks indicate a doublet at nt C18. Black circle in (a) and (b) surrounds the RNAse cleavage sites that are to be fully hindered by other parts of the molecule from any nuclease attack. The nucleotide numbers in (a) and (b) are for the genomic and in (c) for the antigenomic ribozyme. Secondary structure homology between antigenomic and genomic HDV ribozymes [11] was used to project antigenomic RNase cleavage pattern on the structure of genomic ribozyme according to the table shown in Figure 6.

**Figure 6:**
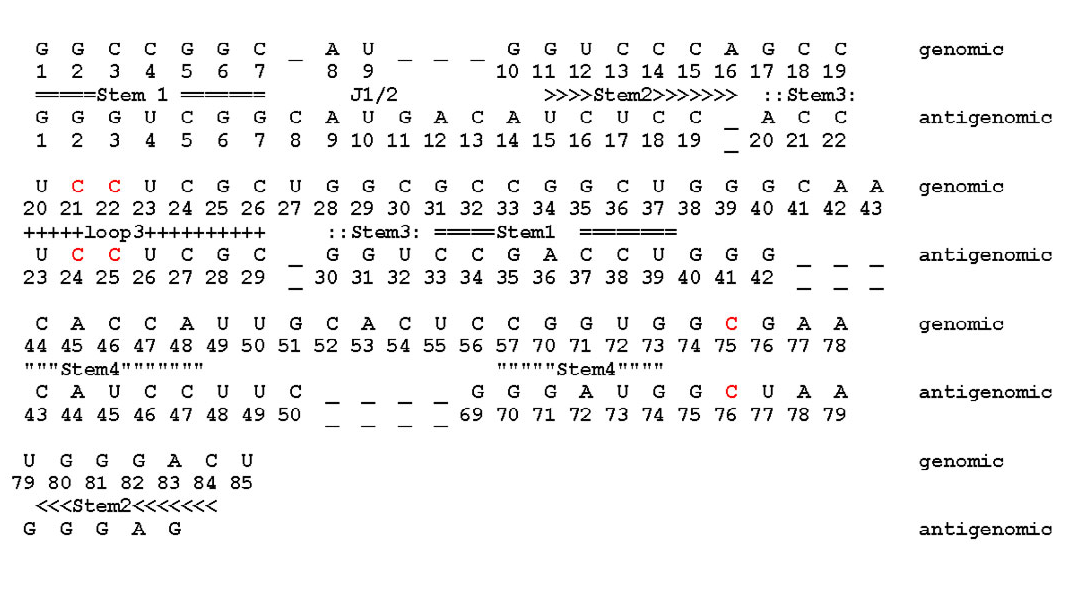
**Alignments of the genomic and antigenomic sequences**. Red letters correspond to nucleotides C21/24, C22/25 of stem P1.1 and catalytic C75/76.

We have projected our nuclease probing data on 3D structures of both ribozyme forms. Atomic coordinates deposited at the RCSB Protein Data Bank (PDB) [10] under accession codes 1drz and 1cx0 for post-cleaved form [2,3] and 1sj3, 1sj4, 1sjf, 1vbx, 1vby, 1vbz, 1vc0, 1vc5, 1vc6 and 1vc7 for pre-cleaved form [4] were used. Secondary structure homology between antigenomic and genomic HDV ribozymes was used accordingly to Been and Wickham alignments of both ribozyme sequences [11] in order to make the projection of our antigenomic data on the structure of genomic ribozyme (Figures 5a and 5b, see Figure 6).

Figure 5a shows a nuclease cleavage digestion pattern obtained for pre-cleaved SBL ribozyme projected on the model of pre-cleaved genomic ribozyme [4], and Figure 5b shows a nuclease cleavage pattern obtained for post-cleaved SBL ribozyme projected on the model of post-cleaved genomic form [2]. For comparison, nuclease probing data of the pre-cleaved SBL ribozyme projected on a 3D model of pre-cleaved SBL are given in Figure 5c. The SBL molecule was modeled on the basis of structural data for HDV ribozyme and optimized with Tripos Sybyl V.6 software run on a Silicon Graphics Computer. For this illustration we have selected a model of pre-cleaved SBL possessing stem P2 which formed without L chain as shown in Figure 3c and composed of nt 16–18 and nt 11–13 instead of nt 82–84 (stem P2'). Several models were considered for a possible conformation of the C16 – U32 region of pre-cleaved ribozyme. The model given in Figure 5c was selected because it shows the best fit for the X-ray data, does not contradict RNAse cleavage pattern, and maintains reasonable orientation of catalytic C76 relative to the cut site phosphate and does not use the 3'end part of L chain for the formation of stem P2. The projection of the RNAse cleavage data on a number of 3D models of antigenomic HDV ribozyme can be found at our laboratory site [6].

As seen from Figure 5a, the major cut sites for both single-strand-specific (red balls) and double-strand-specific (orange balls) nucleases located in the vicinity of the self-cleavage site (green ball) are in conflict with the ribozyme model. The area within black circle in the proximity to C24, C25 (C21, C22 in the genomic ribozyme) contains nuclease cut sites identified by RNAse mapping which are inaccessible to nucleases due to their large size. All other cut sites in this figure and all cut sites in Figure 5b are located at the periphery of the molecule and are accessible to nucleases from the outside. From these data it can be concluded that at least in pre-cleaved antigenomic ribozyme nucleotides C24 and C25 (C21, C22 in the genomic ribozyme) are located on the molecule surface and, consequently, cannot form stem P1.1.

For details and discussion see Additional files 1, 2, 3, 4, 5, 6, 7, 8.

## Conclusion

Dramatic, yet similar changes in the nuclease digestion pattern after the self-cleavage reaction together with the ability of all constructed ribozymes to efficient self-cleavage led us to suggestion that pre- and post-reactive conformations of the ribozyme variants differing in topology and location of cut site have common features, such as the absence of stems P2 and P3 in the form existing in the product and absence of stem P1.1. These stems result from conformational changes during cleavage.

## Competing interests

The authors declare that they have no competing interests.

## Authors' contributions

All experimental procedures were carried out by VA, TB and ND. LS contributed to the design and coordination of the study and helped to draft the manuscript. RB conceived of the study, and participated in the design of the study and performed the data analysis. All authors read and approved the final manuscript.

## Supplementary Material

Additional file 1 Projection of nuclease probing data on 3D structure of ribozymes. Localization of the RNAse cleavage sites on the HDV ribozyme structure 3D models. Click here for file

Additional file 2 Projection of nuclease probing data on 3D structure of pre-cleaved genomic HDV ribozyme. Atomic coordinates and RasMol script directing RasMol program to show nuclease cleavage pattern for pre-cleaved ribozyme projected on its 3D model. All details how to use the rsm files are given in Additional File 1. Click here for file

Additional file 3 Projection of nuclease probing data on 3D structure of post-cleaved genomic HDV ribozyme. Atomic coordinates and RasMol script directing RasMol program to show nuclease cleavage pattern for post-cleaved ribozyme projected on its 3D model. All details how to use the rsm files are given in Additional File 1. Click here for file

Additional file 4 Projection of nuclease probing data on 3D structure of pre-cleaved SBL ribozyme. Atomic coordinates and RasMol script directing RasMol program to show nuclease cleavage pattern for pre-cleaved SBL ribozyme projected on its 3D model. All details how to use the rsm files are given in Additional File 1. Click here for file

Additional file 5 The model of the interaction of single-strand-specific nucleases with the pre-cleaved HDV ribozyme. Atomic coordinates and RasMol script of single-strand-specific nucleases and pre-cleaved HDV ribozyme. All details how to use the rsm files are given in Additional File 1. Click here for file

Additional file 6 The model of the interaction of single-strand-specific nucleases with the pre-cleaved HDV ribozyme. 2D projection of an overlaid structure of single-strand-specific nucleases and pre-cleaved HDV ribozyme. Click here for file

Additional file 7 A comparative analysis of data obtained by RNAse footprinting and X-ray diffraction. The major discrepancy between the ribozyme structures in solution and that obtained upon crystallization. Click here for file

Additional file 8 Material and methods. Enzymes, reagents, preparation of DNA-templates and RNA-product, ribonuclease and Fe(II)-EDTA probing. Click here for file

## References

[B1] Perrotta AT, Been MD (1991). A pseudoknot-like structure required for efficient self-cleavage of hepatitis delta virus RNA. Nature.

[B2] Ferre-D'Amare AR, Zho K, Doudna JA (1998). Crystal structure of a hepatitis delta virus ribozyme. Nature.

[B3] Ferre-D'Amare AR, Doudna JA (2000). Crystallization and structure determination of a hepatitis delta virus ribozyme: use of the RNA-binding protein U1A as a crystallization module. J Mol Biol.

[B4] Ke A, Zhou K, Ding F, Cate JH, Doudna JA (2004). A conformational switch controls hepatitis delta virus ribozyme catalysis. Nature.

[B5] Alekseenkova VA, Belyanko TI, Timofeeva AV, Savochkina LP, Heumann H, Beabealashvilli RSh (2001). Kinetic self-cleavage models for permuted variants of the antigenomic HDV ribozyme. Molekulyarnaya Biologiya (Moscow).

[B6] Properties of antigenomic HDV ribozyme. http://labgen.cardio.ru/en/HDV_ribozyme.

[B7] Rosenstein SP, Been MD (1991). Evidence that genomic and antigenomic RNA self-cleaving elements from hepatitis delta virus have similar secondary structures. Nucleic Acids Res.

[B8] Been MD, Perrotta AT, Rosenstein SP (1992). Secondary structure of the self-cleaving RNA of hepatitis delta virus: applications to catalytic RNA design. Biochemistry.

[B9] Belyanko TI, Alekseenkova VA, Savochkina LP, Lukin MA, Beabealashvilli RSh (2003). Properties of antigenomic hepatitis delta virus ribozyme that consists of three RNA oligomer strands. Biokhimiia (Moscow).

[B10] Berman HM, Westbrook J, Feng Z, Gilliland G, Bhat TN, Weissig H, Shindyalov IN, Bourne PE (2000). The Protein Data Bank. Nucleic Acids Res.

[B11] Been MD, Wickham GS (1997). Self-cleaving ribozymes of hepatitis delta virus RNA. Eur J Biochem.

