# Supplementary material for: RNase footprinting demonstrates antigenomic hepatitis delta virus ribozyme structural rearrangement as a result of self-cleavage reaction
Source: BMC Res Notes. 2008 May 16;1:15. doi: 10.1186/1756-0500-1-15 (PMC2518280; doi:10.1186/1756-0500-1-15)
Supplement: Additional file 1 — Projection of nuclease probing data on 3D structure of ribozymes. Localization of the RNAse cleavage sites on the HDV ribozyme structure 3D models. [file 1756-0500-1-15-S1.doc]

# Additional file 1.

# Projection of nuclease probing data on 3D structure of ribozymes

All the features of our nuclease probing data projected on 3D structures of ribozyme are best seen via rotating 3D models of the both ribozyme forms using Additional files 2 and 3. According to the models, the overall structures of pre-cleaved and post-cleaved ribozymes are almost identical; however, the nuclease cleavage patterns differ dramatically, particularly in the vicinity of nucleotides C21 and C22 which are deeply submerged in the ribozyme body. This is better seen using the model in additional files 5 and 6 which show that large RNAse molecules cannot access the region at nucleotides C21 and C22.

Additional files 2, 3, and 4 correspond to Figure 5a, Figure 5b and Figure 5c of main text respectively. Additional files 2, 3 and 4 provide atomic coordinates of the ribozymes together with RasMol scripts allowing one to view 3D projections of RNAse cleavage pattern on 3D structure of both forms of the ribozyme. Using RasMol or RasTop program one can view the ribozyme structure and RNAse cleavage site projection from different sides. Figure 5 of main text provides a view from one side only. All instructions how to install and use RasMol or RasTop for 3D viewing the data can be found at RCSB site in section “Structure visualization” [1].

Lack of space accessible to nucleases in the vicinity of putative stem P1.1 is illustrated by Additional files 5 and 6. Additional file 5 is a script and coordinate file for a model of the interaction of single‑strand‑specific nucleases with the ribozyme. 3D structure of ribonucleases overlaid on some cut sites of pre-cleaved SBL ribozyme. To model the interaction of single‑strand‑specific nucleases with the ribozyme, we used atomic coordinates and structure factors deposited in the PDB with accession codes: 1bvi, 1rpg and 1goa for RNase T1 (1bvi) [2], for RNAse A (1rpg) [3], while the structure of double‑strand‑specific *E. coli* nuclease H (1goa) [4] was used instead of that of nuclease V1, since its structure is unknown. Additional file 6 is a 2D view of the structure depicted by Additional file 5. It shows that ribozyme and nucleases have similar spatial sizes, implying that the active site and its vicinity in the ribozyme are inaccessible to nucleases. For color code see legend to Figure 5. Although ribonuclease cut sites are put into the active center of the corresponding nuclease, the provided coordinates cannot be considered as a model of the enzyme-substrate complex. It was constructed only to show the relative size of the molecules.

Another feature which is perfectly seen via rotating 3D structures depicted by additional files 2 and 3 is the dramatic difference in the availability of stem 2 of pre-cleaved and post-cleaved ribozyme for V1 nuclease attack. In addition to the suggestion that the structure of a region from 15 ^th^ to 26^th^ nucleotides in pre-cleaved ribozyme is formed without involvement of L chain which was already described in main text, it is noticeable that well exposed stem 2 in post- cleaved ribozyme is available for ribonuclease only in two neighboring sites.

# References

1. **The** **Research Collaboratory for Structural Bioinformatics** [[http://www.rcsb.org/robohelp_f/#molecular_viewers/introduction_to_molecular_viewers.htm](http://www.rcsb.org/robohelp_f/#molecular_viewers/introduction_to_molecular_viewers.htm)]

2. Langhorst U, Loris R, Denisov VP, Doumen J, Roose P, Maes D, Halle B, Steyaert J: **Dissection of the structural and functional role of a conserved hydration site in RNase T1.** *Protein Sci* 1999, **8:** 722-730.

3. Zegers I, Maes D, Dao-Thi MH, Poortmans F, Palmer R, Wyns L: **The structures of RNase A complexed with 3'-CMP and d(CpA): active site conformation and conserved water molecules.** *Protein Sci* 1994, **3:** 2322-2339.

4. Katayanagi K., Miyagawa M, Matsushima M, Ishikawa M, Kanaya S, Ikehara M, Matsuzaki T, Morikawa K: **Three-dimensional structure of ribonuclease H from *E. coli*.** *Nature* 1990, **347:** 306-309.
