# Supplementary figures and images for: RNase footprinting demonstrates antigenomic hepatitis delta virus ribozyme structural rearrangement as a result of self-cleavage reaction
Source: BMC Res Notes. 2008 May 16;1:15. doi: 10.1186/1756-0500-1-15 (PMC2518280; doi:10.1186/1756-0500-1-15)

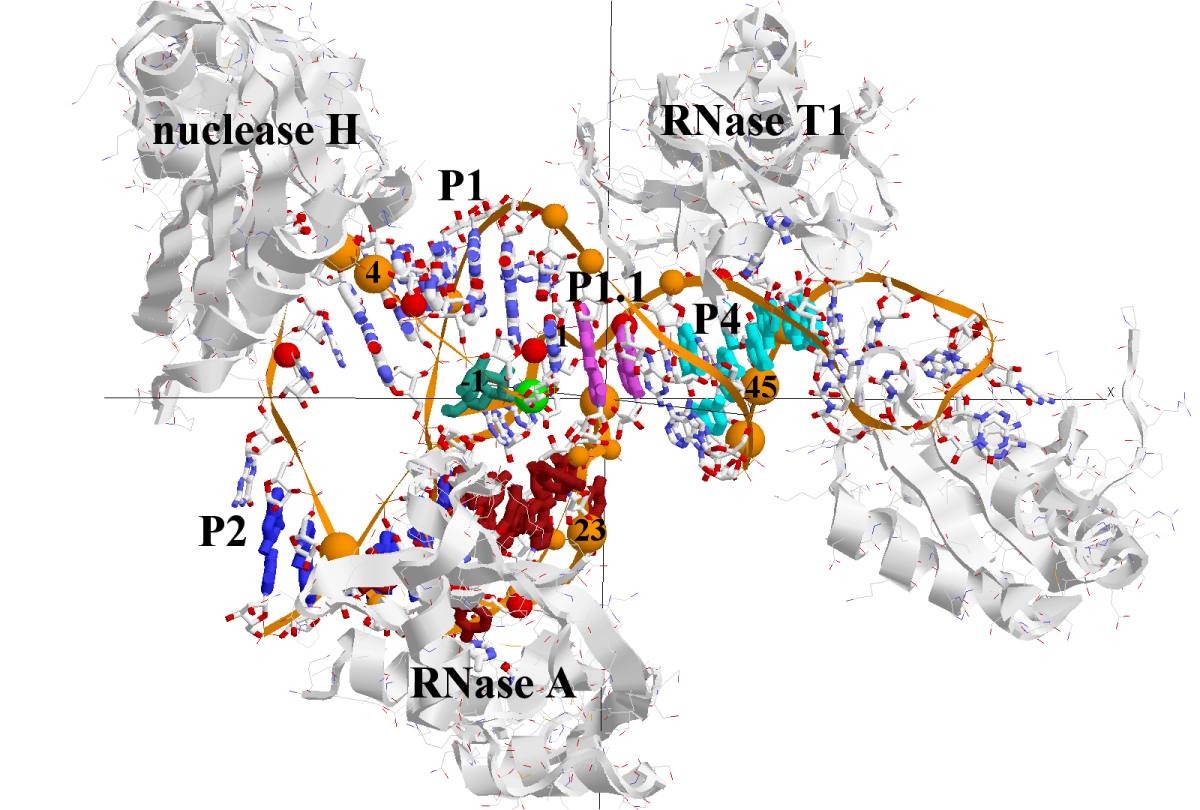

Supplement: Additional file 6 — The model of the interaction of single-strand-specific nucleases with the pre-cleaved HDV ribozyme. 2D projection of an overlaid structure of single-strand-specific nucleases and pre-cleaved HDV ribozyme. [file 1756-0500-1-15-S6.tiff]
