# Supplementary material for: RNase footprinting demonstrates antigenomic hepatitis delta virus ribozyme structural rearrangement as a result of self-cleavage reaction
Source: BMC Res Notes. 2008 May 16;1:15. doi: 10.1186/1756-0500-1-15 (PMC2518280; doi:10.1186/1756-0500-1-15)
Supplement: Additional file 7 — A comparative analysis of data obtained by RNAse footprinting and X-ray diffraction. The major discrepancy between the ribozyme structures in solution and that obtained upon crystallization. [file 1756-0500-1-15-S7.doc]

# Additional file 7

# A comparative analysis of data obtained by RNase footprinting and X-ray diffraction.

The secondary structures of the closely related genomic and antigenomic ribozymes were extensively analysed in the 1990s in Been’s laboratory [1] where four stems were identified and designated P1–P4. The crystal structure of the self-cleaved form of the ribozyme was later solved by Ferre-D’Amare and co-workers [2, 3] and revealed an additional two-base-pair helix termed P1.1. Later, when crystal structure of pre-cleaved genomic HDV ribozyme was solved, it turned out that it is somewhat different from the product form, while the structures of stems P1-P4 and P1.1 are the same in pre- and post-cleave enzyme [4].

Analysis of the ribozyme molecule by various methods has shown that its self-cleavage is accompanied by conformational rearrangements [5-9], which so far remain obscure. We have compared our data with crystal structures of pre- and post-cleaved forms by projecting RNase probing data on 3D models of genomic and antigenomic HDV ribozyme. It turned out that the majority of the nuclease cut sites located in the vicinity of the self‑cleavage site are in conflict with the crystal structure of the pre-cleaved ribozyme published by Ke and co-workers [4]. In the study of Ke and co-workers artificial uncleavable ribozyme was used, and the molecule in a reaction-incompetent conformation was trapped positioning the catalytically essential C75(U75) nucleotide nearby 2’oxygen of C-1, suggesting the general base cleavage mechanism, while from the excellent experiments of Das and Piccirilli [10] it follows that the reaction obeys the general acid mechanism. This unequivocally requires orientation of N3 of C75_g_/C76_a_ nearby 5’ oxygen of G+1 (“a” and “g” as numbered in the antigenomic and genomic forms, respectively).

Since the available data are insufficient, it remains unclear whether these differences result from structural variations in genomic and antigenomic ribozymes (for example, shorter stem P2 in antigenomic ribozyme) or from different structures of the ribozyme in solution and crystalline form. The simplest explanation for the discrepancy seems to come from suggestion that very short (less then 3 nucleotides long) self-cleavage upstream region used for crystallization in study of Ke and co-workers [4] let the molecule to accept the structure forced by crystallization conditions.

If the suggested structure [4] is considered as a catalytically active conformation similar to that adopted by the ribozyme in solution but not as a possible variant of the molecule packing (which is realized upon crystallization), three major controversies will arise:

1. In the structure solved by Ke and co-workers [4] the polynucleotide chain kink at the cleavage site locates the forepart region *f* in the narrow space between stems P1 and P2 and sends it in opposite direction. This should reduce the cleavage rate for LBS, BLS and LSB constructs compared with SBL. However, the activities of all our constructs with an optimal length of linker are practically equal (Figure 1) [11]. In addition, if the described sharp turn at the cleavage site [4] is necessary for the reaction, the minimal length of the linker between the active site and the distal end of stem P2 should be 6 nucleotides, while that to the distal end of stem P4 should be 11-12 nucleotides. Experimentally, in LBS, BLS and LSB these linkers have an equal minimal length of about 9 nucleotides [11].

2. The differences in the nuclease accessibilities of pre- and post-cleaved ribozymes are considerable (Figures 2-4). However, superposition of the structures given previously [3, 4, 9] reveals no difference accounting for the difference in accessibilities to nucleases. The difference in the structure of sugar-phosphate backbone of the corresponding nucleotides for both structures (PDB ID 1sj4 and PDB ID 1drz) expressed in RMSD value is not greater than 1.6 Å, i.e., is comparable to the difference accumulating upon thermal motion during 10 psec of simulation at 300^0^K of each structure. Importantly, the major contribution to RMSD for the sugar-phosphate backbones of pre- and post-cleaved ribozyme is made by U23, C26 and U27 which are not involved in the formation of regular structures in a pre- or post-cleaved ribozyme. Note that the difference in RMSD for the sugar-phosphate backbone of practically identical structures of any of two pre-cleaved forms, for example with PDB ID 1vc7 and 1vbx, are of the same magnitude, namely 0.6 Å.

3. Special attention should be paid to the controversy between the structures formed by nucleotides 16 – 26 in pre- and post-cleaved ribozyme (compare Figures 2a, 2f, 3a, 4a, and 4c with Figures 2b, 3b and 4b). In post-cleaved ribozyme these nucleotides form a chain in stem P2, stem P3 and stem P1.1. There is good agreement between post-cleaved form (PDB ID 1drz and ID 1cx0) [2, 3] and nuclease cleavage pattern for this segment (See Figure 5b and Additional file 3). The structure of this segment in compliance with the coordinates of atoms of uncleaved ribozyme (PDB ID 1sj3, 1sj4, 1sjf, 1vbx, 1vby and 1vbz) does not differ from that in post-cleaved ribozyme (PDB ID 1drz and 1cx0). Both series of data demonstrate the presence of P1.1 that buries nucleotides С24, С25 and their close vicinity into the geometrical center of the molecule which is inaccessible to nucleases. However, digestion patterns for this region are different in uncleaved and product ribozyme forms (compare again Figures 2a, 2f, 3a, 4a and 4c with Figures 2b, 3b and 4b). Moreover, in uncleaved ribozyme this region can be formed without nucleotides 81-84, since the digestion pattern of this region remains the same in the absence and in the presence of L chain (compare Figure 3с with Figures 2a, 2f, 3a, 4a and 4c).

# A tentative scheme of conformational rearrangements upon HDV ribozyme self-cleavage in solution

Experimental analysis of the ribozyme structure in solution is difficult, since the self-cleavage reaction proceeds within a very short time period and small amounts of precursor ribozyme remain in a mixture with the reaction product. This explains at least partially why the structure of uncleaved ribozyme in solution so far remains obscure. All studies have demonstrated conformational changes in individual elements of the ribozyme upon self-cleavage. There are no direct structural data, for example, obtained by NMR, therefore we have to be satisfied with the results of indirect measurements.

Taken together with the kinetic studies of all the ribozyme conformations (including kinetics initiated via fast jumping from different nonpermissive conditions to conditions optimal for the reaction [11]), the data presented here led us to the following hypothesis: the pre-cleaved ribozyme contains stems P2 and P3 in the form other than that in the product and has no stem P1.1. In precleaved form, two cytidine monophospates that participate in the formation of stem P1.1 are located at the periphery of the molecule, which makes them accessible to nuclease V1. In this hypothetical conformation self‑cleavage can proceed but it is fully reversible. Then a relatively slow conformational transition occurs, leading to the formation of a double pseudoknot and removal of the region *f* from the reaction zone. This reaction is reversible once conformational transitions in the molecule led to the formation of stem P1.1, which secures the results of self-cleavage. This transition makes reaction practically irreversible due to formation of a very tight structure with stems P2 and P1.1 being the major source of its stability. The scheme provides the simplest explanation for the two‑phase kinetics of the reaction, great conformational differences in pre- and post-cleaved ribozyme, and self-cleavage irreversibility which is independent of the dissociation efficiency of the reaction products.

In conclusion, the nuclease cleavage patterns of SB product of LSB self‑cleavage and the patterns of pre-cleaved forms of all topological variants are similar, indicating that SB forms an L‑independent domain in uncleaved ribozyme and can be used as a model to study the structure of SB. The LB product of LBS or BLS self‑cleavage is similar to the product of SBL cleavage and can be used to study major structural features of the final form of the reaction product. Both fragments are small enough and not reactive, which makes them suitable for NMR analysis.

# References

1. Perrotta AT, Been MD: **A pseudoknot-like structure required for efficient self-cleavage of hepatitis delta virus RNA.** *Nature* 1991, **350:** 434-436.

2. Ferre-D’Amare AR, Zho K, Doudna JA: **Crystal structure of a hepatitis delta virus ribozyme.** *Nature* 1998, **395:** 567-574.

3. Ferre-D'Amare AR, Doudna JA: **Crystallization and structure determination of a hepatitis delta virus ribozyme: use of the RNA-binding protein U1A as a crystallization module.**  *J Mol Biol* 2000, **295:** 541-556.

4. Ke A, Zhou K, Ding F, Cate JH, Doudna JA: **A conformational switch controls hepatitis delta virus ribozyme catalysis.** *Nature* 2004, **429:** 201–205.

5. Harris DA, Rueda D, Walter NG: **Local conformational changes in the catalytic core of the trans-acting hepatitis delta virus ribozyme accompany catalysis.** *Biochemistry* 2002, **41:** 12051–12061.

6. Pereira MJ, Harris DA, Rueda D, Walter NG: **Reaction pathway of the trans-acting hepatitis delta virus ribozyme: a conformational change accompanies catalysis.** *Biochemistry* 2002, **41:** 730–740.

7. Tanaka Y, Tagaya M, Hori T., Sakamoto T., Kurihara Y, Katahira M, Uesugi S: **Cleavage reaction of HDV ribozymes in the presence of Mg2+ is accompanied by a conformational change.** *Genes Cells* 2002, **7:** 567–579.

8. Jeong S, Sefcikova J, Tinsley RA, Rueda D, Walter NG: **Trans-acting hepatitis delta virus ribozyme: catalytic core and global structure are dependent on the 5’ substrate sequence.** *Biochemistry* 2003, **42:** 7727–7740.

9. Harris, DA, Tinsley RA, Walter NG: **Terbium-mediated footprinting probes a catalytic conformational switch in the antigenomic hepatitis delta virus ribozyme.**  *J Mol Biol* 2004, **341:** 389–403.

10. Das SR, Piccirilli JA: **General acid catalysis by the hepatitis delta virus ribozyme.** *Nat Chem Biol* 2005, **1**(1)**:** 45-52.

11. Alekseenkova VA, Belyanko TI, Timofeeva AV, Savochkina LP, Heumann H, Beabealashvilli RSh: **Kinetic self-cleavage models for permuted variants of the antigenomic HDV ribozyme.** *Molekulyarnaya Biologiya (Moscow)* 2001, **35:** 750-768 (*(English Translation*, [*SpringerLink.com*](http://www.springerlink.com/content/k32424n2497n8811/?p=8b13e8588b3c4357b6feb33dbf1523a3&pi=1)).
