# Supplementary material for: RNase footprinting demonstrates antigenomic hepatitis delta virus ribozyme structural rearrangement as a result of self-cleavage reaction
Source: BMC Res Notes. 2008 May 16;1:15. doi: 10.1186/1756-0500-1-15 (PMC2518280; doi:10.1186/1756-0500-1-15)
Supplement: Additional file 8 — Material and methods. Enzymes, reagents, preparation of DNA-templates and RNA-product, ribonuclease and Fe(II)-EDTA probing. [file 1756-0500-1-15-S8.doc]

# Addition file 8

# Materials and Methods.

## Enzymes and reagents

Ribonucleases (RNase) T1, V1 were obtained from Ambion (TX, USA), U2 was from Pierce (WI, USA), RNase A was from Sigma-Aldrich (MO, USA), T7 RNA-polymerase and reagents for transcription were from Promega (WI, USA), Taq DNA-polymerase, T4 RNA ligase, DNA ligase and polynucleotide kinase were from Fermentas (Lithuania). Oligonucleotides were synthesized in a Beckman System 1 Plus DNA Synthesizer. The [α-^32^P]UTP and [γ-^32^P]ATP with a specific activity of 2000-5000 Ci/mmole were from Isotop (Moscow, Russia). NenSorb 20 columns were purchased from Perkin Elmer (MA, USA).

## Preparation of DNA-templates

DNA-templates for HDV ribozyme analogs were prepared by joining complementary and overlapping oligodeoxyribonucleotides which represent the entire length of the ribozyme, and T7 RNA promoter. A mixture containg 50 mM Tris-HCl, pH 7.5, 10 mM MgCl_2_, 0.1 mM spermidine, 0.1 mM EDTA, 5 mM DTT and 2 µM each oligonucleotide was incubated at 85^0^C for 2 min, slowly cooled to 37^0^C and incubated with T4 DNA ligase for 120 min. The resulting DNA was fractionated by electrophoresis in a 10% polyacrilamide/7 M urea gel. DNA was eluted from the band corresponding to the full-length product with 1 mM EDTA overnight at 4^0^C, purified on a Nensorb-20 column, dried *in vacuo* and dissolved in nuclease-free H_2_O. Then DNA was ligated to HindIII/SmaI codigested pUC19 yielding a plasmid harboring the ribozyme. To verify the design all the DNAs were sequenced by PCR with 3’F‑ddNTP chain-termination [1].

## Preparation of RNA-product

RNA was synthesized by T7 RNA polymerase runoff transcription reaction in a mixture containing 80 mM HEPES-KOH (pH 7.5), 24 mM MgCl_2_, 2 mM Spermidine, 10 mM DTT, 0.5 -1 µM DNA‑template, rNTPs at 1 mM each, 4 mM GpG, and 30 units/µl T7 RNA polymerase. Radiolabeled transcripts were prepared with [α-^32^P]UTP included in the reaction. Typically, the *in vitro* transcription reaction was carried out at 20^0^C for 60 min, the products were phenol extracted, ethanol precipitated and fractionated in 8 % polyacrylamide/ 7 M urea gel. Individual RNA bands corresponding the full‑length RNA or cleavage product were located by UV shadowing or autoradiography, eluted overnight at 4^0^C in 1 mM EDTA and chromatographed on a Nensorb 20 column. RNA’s were dried *in vacuo*, dissolved in nuclease-free water and stored at -20^0^C. RNA concentration was determined from light absorbance measurement or from the specific activity of UTP, UTP content of each RNA molecule and radioactivity of RNA fragment.

## Selective labeling of RNA transcripts

Gel-purified RNA was labeled at the 5’end with T4 polynucleotide kinase and [γ-^32^P]ATP [2] and at the 3’end with [5’-^32^P]pCp and T4 RNA ligase [3] except that both reactions were performed at 20^0^C for 15-45 min, depending on RNA self-cleavage activity. Labeled RNA was gel purified as described above. The sequence of the RNA was verified by limited enzymatic digestion with RNases T1, A and U2 at 50^0^C in a buffer with pH 3.5 and 7 M urea [2].

## Ribonuclease probing and Fe(II)-EDTA assays

Structure probing reactions (10 µl volume) with RNase T1, U2 or A contained 20,000 cpm (Cherenkov) of end-labeled RNA, 25 mM Tris-HCl, pH 7.5, 200 mM NaCl, 0.2 mg/ml yeast tRNA, 10 mM MgCl_2_ and were performed at 20^0^ for 5 min (under the conditions minimizing self-cleavage reaction). To generate sequencing markers RNA was incubated with RNases in a buffer containing 20 mM Na-citrate, pH 3.5, 1 mM EDTA, 0.2 mg/ml yeast tRNA and 7 M urea at 50^0^C. The final concentration of RNase V1 in each reaction was 1x10^-3^ or 5x 10^-3^ units/µl, RNase T1 - 2x10 ^-3^ units/µl, RNase A - 1x10^-5^ µg/µl, RNase U2 - 5x10^-2^ units/µl at 20^0^C and 1.7x10^-2^ units/µl at 50^0^C. After a 5-min incubation, 1 µl mixture containing 100 mM EDTA, 3 M NaAc, pH 4,5 was added, followed by addition of 33 µl EtOH, and the reactions were put on crushed dry ice for 15-30 min. The pellets were dissolved in a mixture containing 12.5 mM EDTA, 45% formamide, 0.02% each bromphenol blue and xylene cyanol, heated at 95^0^C for 2 min and loaded on 12% polyacrylamide gel (20:1 acrylamide: bisacrylamide) in 0.09 M Tris-Borate, pH 8.4, 2.5 mM edta, 7 M urea. After electrophoresis the gel was exposed to X-ray film at -70^0^C overnight.

## Hydroxyl radical cleavage

Hydroxyl radical cleavage (Fe(II)-EDTA) of 5’‑labeled RNA was carried out by the method of Celander and Cech [4]. The sample was then treated and analyzed as in the case of ribonucleases probing.

# References

1. Savochkina LP, Diachenko LB, Lukin MA, Aleksandrova LA: **Analogs of nucleotides, modified by a sugar residue and pyrimidine base, in a DNA synthesis reaction, catalyzed by *Thermus aquaticus* DNA polymerase.** *Molekulyarnaya Biologiya  (Moscow)* 1992, **26:** 191-200 *(English Translation)*.

2. Rosenstein SP, Been MD: **Evidence that genomic and antigenomic RNA self-cleaving elements from hepatitis delta virus have similar secondary structures.** *Nucleic Acids Res* 1991, **19:** 5409-5416.

3. England TE, Bruce AG, Uhlenbeck OC: **Specific labeling of 3’termin of RNA with T4 RNA ligase.** In *Methods Enzymol. Volume* *65.* Edited by Colowick SP, Kaplan NO. New York, Academic Press; 1980: 65-74.

4. Celander DW, Cech TR: **Visualizing the higher order folding of a catalytic RNA molecule.** *Science* 1991, **251:** 401-407.
